# Friendship Network and Dental Brushing Behavior among Middle School Students: An Agent Based Modeling Approach

**DOI:** 10.1371/journal.pone.0169236

**Published:** 2017-01-19

**Authors:** Maryam Sadeghipour, Mohammad Hossein Khoshnevisan, Afshin Jafari, Seyed Peyman Shariatpanahi

**Affiliations:** 1 Preventive Dentistry Research Center, Research Institute of Dental Sciences, Shahid Beheshti University of Medical Sciences, Tehran, Iran; 2 Community Oral Health Department, School of Dentistry, Shahid Beheshti University of Medical Sciences, Tehran, Iran; 3 Faculty of Management, University of Tehran, Tehran, Iran; 4 Institute of Biochemistry and Biophysics, University of Tehran, Tehran, Iran; Cinvestav-Merida, MEXICO

## Abstract

By using a standard questionnaire, the level of dental brushing frequency was assessed among 201 adolescent female middle school students in Tehran. The initial assessment was repeated after 5 months, in order to observe the dynamics in dental health behavior level. Logistic Regression model was used to evaluate the correlation among individuals’ dental health behavior in their social network. A significant correlation on dental brushing habits was detected among groups of friends. This correlation was further spread over the network within the 5 months period. Moreover, it was identified that the average brushing level was improved within the 5 months period. Given that there was a significant correlation between social network’s nodes’ in-degree value, and brushing level, it was suggested that the observed improvement was partially due to more popularity of individuals with better tooth brushing habit. Agent Based Modeling (ABM) was used to demonstrate the dynamics of dental brushing frequency within a sample of friendship network. Two models with static and dynamic assumptions for the network structure were proposed. The model with dynamic network structure successfully described the dynamics of dental health behavior. Based on this model, on average, every 43 weeks a student changes her brushing habit due to learning from her friends. Finally, three training scenarios were tested by these models in order to evaluate their effectiveness. When training more popular students, considerable improvement in total students’ brushing frequency was demonstrated by simulation results.

## Introduction

Oral health as an important component of general health plays a significant role in individuals’ overall health, wellbeing and quality of life. Oral diseases are highly prevalent despite being preventable. One of the most effective ways to improve oral health status of the society is to change or modify people’s life styles, specifically their oral health behaviors. Based on dental public health principles, dental health behavior can be influenced by many different factors such as those of general health [1–3] as well as psychological [4], socio-economical [5] and environmental factors[6]. Dealing with such multifactorial conditions is not easy when considering such complexities. Under such circumstances, using social networks, as an effective tool on individuals’ lifestyle, may potentially be considered as a new approach [7]. Improvement in community oral and dental health promotion was the main objective of this approach. It has been suggested that, one’s attitudes, beliefs and health behaviors, are deeply influenced by his/her peer connections in one’s social network. In this case, we assumed that, these facts can be utilized as effective tool to promote healthy lifestyle.

There are several reports suggesting positive effects of social network on public health issues in different areas [8, 9]. Pinquarts et al. reported a clear positive association after reviewing 286 articles on social network and health [10]. Other investigations have indicated that, social networks have been helpful in reducing risk of mortality [11, 12]. Also, positive relationship has been reported between social contacts and mental health[13, 14]. Likewise, social network has been found to be helpful in improving the health related behaviors in subgroups of society [15] like alcohol users [16, 17] people with obesity [18] and certain sexual behavior [19]. The important effects of social network on individuals’ health status becomes more evident when we observe internet based social networks are rapidly expanding and playing very effective role in people’s everyday living. These studies envision a promising future for health related behavior and knowledge (health literacy) promoting through social networks [20, 21].

By definition, Agent Based Modeling is a computational method that can be used to evaluate collective behaviors in different social structures. It models each individual’s behavior and considering its interaction with other agents. In addition, ABM can account for the environmental circumstances and its influence on the society’s given status [22]. Based on simple rules, ABM is able to predict different patterns and behaviors at the societal level and their emergent properties [23]. While these behaviors and properties are hidden behind the interactions among the model elements, they can be uncovered through model simulation. In contrast to other system modeling methods, such as system dynamics, ABM focuses on the agents’ relations through social networks and indicates that in many cases the network structures can have significant effects on the patterns of behaviors at societal level. In short, ABM is most useful when the dynamics of behaviors at societal level is deeply influenced by individuals’ interactions through social networks.

Although, there are only a few reports of ABM applications on oral and dental public health [24–26], the fast growing application of this method in other public health areas are evident.

Originally, ABM was introduced as a new tool to understand different epidemiological problems [27–29]. It was specially employed to investigate on problems associated with spread of contagious diseases [30–36]. Likewise, it was used to investigate health related behaviors [15, 16, 18, 19]. Also, some studies focused on spreading of health related emotions in society [37, 38].

This paper can be considered as one of the pioneering works investigating the dynamics of individual’s oral health behavior, specifically tooth brushing, in a social network using ABM. In addition, the model results are compared to an empirical data on adolescents in a middle school setting. The target society was composed of adolescents whom were shown to have very effective social network that had influence on their health behaviors [39, 40]. The proposed mechanisms for the behavior diffusion through the network are students’ indirect learning from their friends. This learning style can initiate by simple observations such as halitosis (bad breath) and/or attractive smile (beautiful teeth).

## Materials and Methods

### First round of assessment

A standard questionnaire was developed to assess the relationship between oral health related behavior and their social network structure. A questionnaire was given to 201 female students, who were between 12–13 years old. The participants were first year middle school students from the same school, attending 7 different classrooms, each containing about 30 students. The assessment was performed over November 2014.

In first step, standardization of questionnaire was conducted by using ten dental public health and sociology experts to assess the validity of each question using qualitative and quantitative methods. In addition, the face validity for each question was calculated. Finally, test–retest reliability was conducted which showed kappa coefficient values of greater than 0.75.

The questionnaire was composed of 8 questions for assessment of socioeconomic status (SES) of students’ family by using parental education level and their economic status.

Oral health behavior was assessed by asking about the frequency of tooth brushing. Four-level scoring system was used: Two times or more per day (1), Once daily (2), Few times per week (3) and never (4).

Students were asked to list up to five names of their close friends. Consequently, a 201 X 201 friendship matrix was formed, containing the information of each individual’s friends, so that there was a specific row and column for each individual. The matrix element *x*_*ij*_, denoting row *i* and column *j*, denoted as 1 if student *i* had named student *j* as her friend otherwise assigned as Zero “0”. For subsequent levels of friendship (levels 2 or more), the relevant matrix indicated indirect friendship relations mediated by 1 or more students respectively. In other words *level n* friendship matrix shows whether two students have a distance of n links in the network. This *level n* friendship matrix can be formed by multiplying the first level friendship matrix n times by itself. Calculations at this stage were handled by using R programming language.

### Second round of assessment

In order to assess the dynamics of brushing behavior within the social network, the same questions were asked from students after laps of 5 months. At this time, the same 201 students participated in the second round of assessment. It should be noted that, the questions on SES were omitted in the 2^nd^ assessment, by the assumption that, nothing has been potentially changed over the past 5 months.

### Statistical analysis

A logistic regression analysis was implemented for modeling the effects of social network on adolescents’ oral health behavior, while controlling for the effect of SES. This analysis was used to identify the relationship between brushing frequency among adolescents and their social network structure after adjusting for other interfering variables.

Furthermore, the correlation between individuals’ brushing behavior was calculated, within specific friendship distance in the network. For doing so, the following vectors were used: individuals’ brushing frequency *(V*_*0*_*)*, 1^st^ level friends’ brushing frequency *(V*_*1*_*)*, 2^nd^ level friends’ brushing frequency *(V*_*2*_*)* as well as subsequent levels. The cross correlations of the *i*^*th*^ level vector (*V*_*i*_) and *V*_*0*_ were calculated. These values can identify the correlation between individuals’ behaviors at different levels of friendship.

In addition, the correlation between ones’ popularity and her frequency of brushing was assessed using logistic regression model. Students’ In-degree value was considered as a popularity measure. One’s in-degree is found, by definition, by counting the number of students who have named her as friend. The use of this measure was based on some of the previous works in social network literature proposing the “Degree of centrality” as a popularity measure of nodes in a network [41–44] where each node stands for a student in this analysis. This definition is somehow analogous to the definition of socio-metric popularity introduced in sociology context. The socio-metric popularity, in contrast to perceived popularity, refers to liked people with nice friendship behaviors while, perceived popular individuals are well-known for their dominance and are emulated [45]. Transmission of the dental brushing behavior was assumed to be unintentional and indirect through practical reasons such as halitosis (bad breath) and/or nice smile (beautiful teeth). Students need to spend some time with each other in order to learn or modify certain behaviors or habits. Therefore, it seems reasonable to define the “degree of centrality” as a value and measure of popularity in social friendship network related to the tooth brushing behavior.

### Modeling

In this investigation the ABM was used to demonstrate dynamics of brushing behavior among students in social network. Two series of simulations, relevant to two different models, were performed as follows:

First assuming that, agents were connected through a static network. In this case, the network structure was set in the same way as it was extracted from the 1^st^ round of the empirical study.

**Fig 1:**
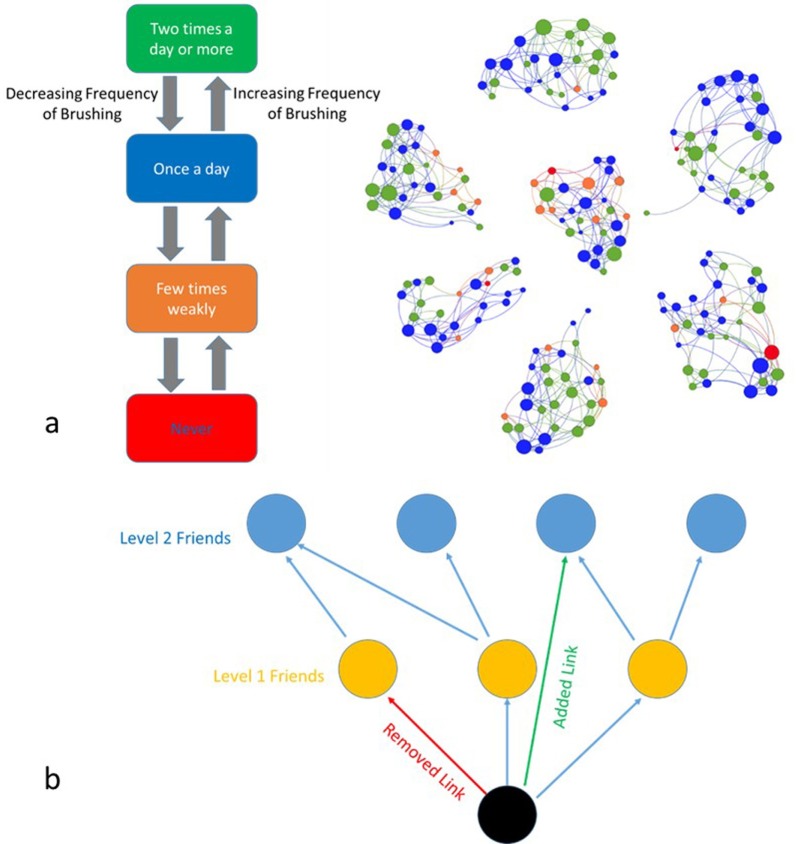
Schematic of the Agent Based Model. **(a)** Four different status of agent’s brushing frequency. Nodes’ color shows the level of the agent’s brushing frequency of brushing and node’s size shows her popularity. **(b)** Changing a student’s friendship relations in the network (dynamic network model).

Fig ***1***(A) shows the network structure in 7 classrooms found in the 1^st^ round of empirical results. Agents demonstrated four levels of brushing frequency habit relevant to the four levels of brushing frequency in the empirical study. Here, it was assumed that, every week, each student with a probability *u* decides to change her level of brushing frequency by one level in order to get closer to the average level of her friends. It should be noted that such behavior-change by average friends as a simple model was adopted from previous studies on spreading of alcohol use behavior [16, 17], opinion formation [46] and learning [47] through social networks. It should also be mentioned that, since the first assessment was performed just two months after the start of school year and the friendship connections among students were just recently formed the network was not in steady state at the period of time of the empirical assessments.

In order to calibrate the model, we compared the speed of spreading of behavior through network for empirical and experimental results. The spreading speed can be measured by calculating the speed of behavior correlation diffusion through the network. To do so, the simulation results of first assessment were used as initial conditions, and the correlation of tooth brushing frequency in the second round of empirical assessment was compared to simulation results for different levels of friendship. For simulation results, the correlation was determined by finding *i*^*th*^ level of friends’ brushing frequency vectors (V0, V1, V2, …) and averaging over the results of 100 times iterated simulations. Then, the cross correlation values of brushing levels for friends, at different distance in the network, were calculated as follows:

$${CC}_{i}=\sum_{j}^{N}V_{0}{(j)V}_{i}(j)$$

Where CC_i_ was the correlation of individuals’ brushing frequency with their i^th^ level of friends and N was the total number of students. When comparing these values with relevant values from the empirical results, we could identify the probability of changing behavior weekly (*u*) for the best fit.

In the second series of simulations, the network structure was considered to be changing over time. We assumed that each week, a student with probability *u* decides to change her brushing frequency level. In addition she also decides whether to change one of her friends with probability *p*. In this process, she removes one of her friendship connection randomly and adds on a new direct connection to another random student with 2 distances from her.

Fig ***1***(B) shows this substitution. To find probability *p* for different *p* values, the difference between the simulations results for level 1 friendship matrices at initial time and after 5 months were calculated. This difference was compared with relevant difference of the empirical data, for 1^st^ and 2^nd^ round assessment in order to identify the best *p* to replicate the empirical results. Finally, *V*_*0*_, *V*_*1*_, *V*_*2*,...._were calculated and the model was calibrated with same process as described for the static network model.

In order to illustrate some applications of the developed model, three training scenarios were also simulated:

“No Training” scenario: The dynamic network model was simulated using the first round empirical results as initial values of dental brushing level. Number of students with brushing level of “twice a day or more” was used as an indicator of students’ optimum brushing level.

“Random Training” scenario: starting with the first round empirical results, using 10 randomly selected students for training on “twice a day or more” brushing principal. The simulation was run based on modified levels of brushing values.

“Popular Selected Training” scenario: starting with the first round empirical results, using 10 students, with highest degree of popularity. They were trained on “twice brushing a day or more” principal. The simulations were continued using modified levels of brushing values.

All simulation results were reported after 100 iterations over time on average.

The parental written consent was obtained prior to student’s participation and the study protocol was approved by the “Research Ethics Committee” of the Research Institute of Dental Sciences at the School of Dentistry, Shahid Beheshti University of Medical Sciences. Tehran, Iran.

## Results

### Empirical Results

The frequency distribution of daily tooth brushing in the first round assessment showed that 39.3% of the students brushed two or more times daily; about 48.2% of students brushed once a day and the rest (12.3%) reported sometimes in a week or no brushing (Table 1). While, in the second round of assessment 48.7% of the students brushed their teeth two or more times daily; about 43.7% of students brushed once a day and the rest (7.6%) reported sometimes in a week or no brushing (Table 1). The average score for the first and second rounds brushing level were 1.75 ± 0.01 and 1.61 ± 0.01 respectively (consider that the scores decrease as the brushing frequency increases). This data indicates that, the level of brushing frequency has somehow improved compared to the first round assessment.

**Table 1:** Frequency analysis of students with different levels of brushing frequency.

Bushing Frequency Levels	Score	First round assessment		Second round assessment	
		Frequency	Percent	Frequency	Percent
**twice or more**	1	79	39.3	98	48.7
**once**	2	97	48.2	88	43.8
**some times in a weak**	3	21	10.4	11	5.5
**no brushing**	4	4	2	4	2
**Total**		201	100.0	201	100
**Score average**		1.75 ± 0.01		1.61 ± 0.01	

Table 2 indicates some of the statistical characteristics of the students’ friendship relations. Each student has listed almost 4 names, on average, and about 35% of the students are also listed by their friends. Furthermore, Table 2 shows the average score of brushing for the students’ friends. When comparing these results with Table 1, we find that, on average, ones’ friends have better brushing status than the student herself.

**Table 2:** Frequency analysis of statistics characterizing the friendship relations.

Parameter	Value	First Round		Second Round	
		Frequency	Percent	Frequency	Percent
**Number of friends named by student (Nodes’ out-degree)**	**0**	11	5.5	13	6.4
	**1**	0	0	1	0.5
	**2**	8	4	7	3.5
	**3**	34	17	23	11.5
	**4**	41	20.3	60	29.9
	**5**	107	53.2	97	48.2
**Average of the number of friends**		4.06 ± 0.09		4.02 ± 0.09	
**Percentage of a students who mutually named each other as friends**	**0**	34	16.9	44	21.9
	**20**	53	26.4	54	26.9
	**40**	57	28.3	38	18.9
	**60**	43	21.4	39	19.3
	**80**	8	4	20	10
	**100**	6	3	6	3
**Average percentage of mutual friendships**		35.6 ± 2.4		35.5 ± 2.4	
**Average brushing score of the students’ friends**		1.68 ± 0.02		1.6 ± 0.02	

In order to characterize the observed change in the social network from first to the second assessment, we summed up the elements of difference for the first and second empirical friendship matrices using the following formula:

$$D=\sum_{i,j}^{N}\left|Y_{ij}-X_{ij}\right|=628$$

Where X and Y were the friendship matrices obtained from the first and second assessments. Considering that on average, each student has named almost 4 others as her friends, the above value of D = 628 was about 40% of the maximum possible changes of 201*8 = 1608; where 8 was the maximum value for a student list of change when she changes all her 4 friends.

After controlling for effect of SES, there was a positive relationship between an adolescent’s daily brushing frequency and average brushing of her friends’ (*p-value<0*.*05*) for both rounds of assessments (Table 3). While an adolescent’s daily tooth brushing was not significantly related with average tooth brushing of her friends of friends (level 2) in the first round, a significant correlation was observed for the second round (*p-value<0*.*05*). It should be noted that, for the subsequent levels of friendship no relationship was observed in either rounds.

**Table 3:** Logistic regression model for the relation between one’s brushing frequency and average of her friends (with distance 1 and 2) after controlling for the effect of socioeconomic status.

Round	Friend-ship Level	Parameter		Estimate(B)	Std. Error	95% Wald Confidence Interval		Hypothesis Test			Exp.(B)	95% Wald Confidence Interval for Exp. (B)	
						Lower	Upper	Wald Chi-Square	df.	Sig.		Lower	Upper
1	1	Level of brushing Threshold	1	-0.472	0.7993	-2.039	1.094	0.349	1	0.554	0.623	0.130	2.987
			2	2.167	0.8171	0.566	3.769	7.036	1	0.008	8.736	1.761	43.334
			3	3.566	0.8870	1.828	5.305	16.168	1	0.000	35.389	6.221	201.302
		Economy		-0.017	0.0927	-0.198	0.165	0.032	1	0.858	0.984	0.820	1.179
		Education		-0.283	0.1031	-0.485	-0.081	7.548	1	0.006	0.753	0.615	0.922
		Average of friends		0.439	0.2209	0.006	0.872	3.950	1	0.047*	1.551	1.006	2.392
		(Scale)		0.659									
	2	Level of brushing Threshold	1	-0.823	0.8260	-2.442	0.796	0.992	1	0.319	439	0.087	2.217
			2	1.793	0.8399	0.147	3.440	4.560	1	0.033	6.010	1.159	31.174
			3	3.185	0.9087	1.404	4.966	12.283	1	0.000	24.160	4.070	143.406
		Economy		0.001	0.0921	-0.179	0.182	0.000	1	0.990	1.001	0.836	1.199
		Education		-0.290	0.1027	-0.491	-0.088	7.953	1	0.005	0.749	0.612	0.915
		Average of friends of friends		0.135	0.2352	-0.326	0.596	0.331	1	0.565	1.145	0.722	1.815
		(Scale)		0.659									
2	1	Level of brushing Threshold	1	1.906	0.8491	0.242	3.570	5.038	1	0.025	6.725	1.273	35.517
			2	4.441	0.8883	2.700	6.182	24.994	1	0.000	84.839	14.877	483.825
			3	6.436	.9799	4.516	8.357	43.142	1	0.000	624.12	91.446	4259.71
		Economy		0.323	0.1005	0.126	0.520	10.308	1	0.001	1.381	1.134	1.681
		Education		-0.134	0.1043	-0.338	0.070	1.650	1	0.199	0.875	0.713	1.073
		Average of friends		0.571	0.2305	0.120	1.023	6.145	1	0.013*	1.771	1.127	2.782
		(Scale)		0.735									
	2	Level of brushing Threshold	1	1.814	0.8581	0.132	3.496	4.467	1	0.035	6.133	1.141	32.969
			2	4.329	0.8933	2.578	6.080	23.486	1	0.000	75.872	13.174	436.962
			3	6.312	0.9792	4.393	8.231	41.552	1	0.000	551.15	80.867	3756.39
		Economy		0.341	0.0983	0.148	0.533	12.022	1	0.001	1.406	1.160	1.705
		Education		-0.133	0.1020	-0.333	0.067	1.706	1	0.192	0.875	0.717	1.069
		Average of friends of friends		0.422	0.2275	-0.024	0.867	3.435	1	0.044*	1.524	0.976	2.381
		(Scale)		0.706									

* Significant (*p-value<0*.*05*)

The correlation length over the network, is a measure indicating the maximum distance in friendship network that two individuals’ brushing frequency level are correlated. The correlation length in the first round assessment was 1 as one’s brushing level was correlated with her direct friend, while the correlation length in the second round was 2 as individuals’ brushing behavior were correlated up to level 2 friends. It indicates that the correlation length, in the network, increases after 5 month. This fact shows that the system is not in the steady state during the period between the two assessments.

**Fig 2:**
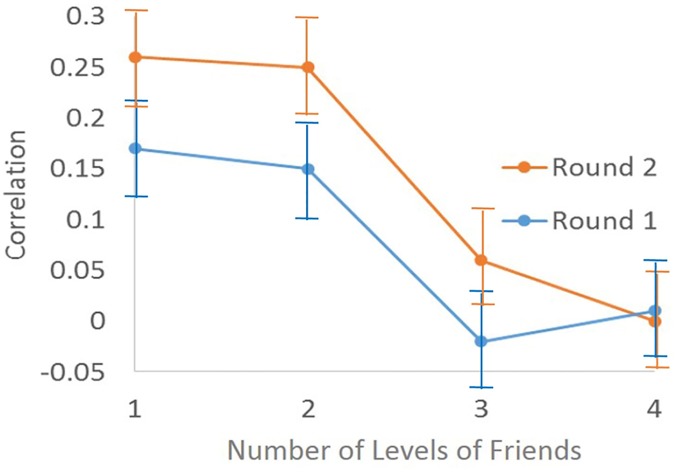
Correlation between agents’ brushing frequency and their friends in different levels of friendship (distance in the network).

Fig 2 shows the cross correlation between students’ level of brushing frequency and her distant friends in the network (different levels of friendship). As expected, the correlation decreases as the friendship level (distance in the network) increases. In other words, closer agents in the network are more correlated in their level of brushing. Interestingly, correlation values of the first round were higher than the second round; admitting the fact that, the social network among students affects their dental health behavior. These results were analogous to the logistic regression model results for the increment of the correlation length in the network (Table 3).

In addition, bivariate logistic regression model was used to find correlation between one’s brushing frequency and her popularity (node’s in-degree). Students’ popularity was measured by the number of students who have named her as their friend. Table 4 shows the model coefficients, indicating a significant relationship between the popularity and her level of brushing frequency. The regression model showed that, the popularity was higher as the brushing frequency level was more frequent (less score).

**Table 4:** Logistic regression model for the relationship between an agent’s brushing frequency level and the degree of her incoming connections, results are based on the data from the first round of assessment.

Parameter		Estimate (B)	Std. Error	95% WaldConfidence Interval		Hypothesis Test			Exp. (B)	95% Wald Confidence Interval for Exp. (B)	
				Lower	Upper	Wald Chi-Square	df	Sig.		Lower	Upper
Threshold	1	-0.987	0.3180	-1.611	-0.364	9.643	1	0.002	0.373	0.200	0.695
	2	1.443	0.3385	0.780	2.107	18.180	1	0.000	4.235	2.181	8.223
	3	3.408	0.6016	2.229	4.587	32.091	1	0.000	30.200	9.289	98.190
Popularity (Node’s in-degree)		-0.141	0.0696	-0.277	-0.004	4.078	1	0.043*	0.869	0.758	0.996
(Scale)		1.205									

* Significant (*p-value<0*.*05*)

### Model Calibration

**Fig 3:**
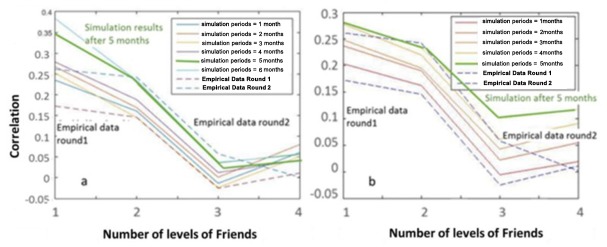
Comparison of the cross correlation values for different friendship level of empirical data and simulation. (a) static network model and (b) dynamic network model. The u, as probability of changing an agent’s brushing frequency habit in a week, was assumed to be 0.02 and 0.023 for static and dynamic network model respectively.

In order to calibrate the first model (to find *u* in static network) we focused on the cross correlation of agents’ frequency of brushing and their friends’ with different distances in the network (different friendship levels). As shown in **Fig 3**(A), for the static network model, *u* value were chosen in a way that, the correlation diagram of the empirical data for the second round best fits to the simulation results after 5 months. The simulated 5^th^ month result is indicated with a thick green line in the figure which should be compared to the blue dashed line indicating to the second round empirical data. The best fit results in *u = 0*.*02* meaning that every 50 weeks, one student decides to change her habit to become more similar to her friends. As the figure illustrates, simulation results does not fit well to the empirical data for this model.

To calibrate the second model with dynamic network structure, we first found *p* by comparing the difference between the 2^nd^ and 1^st^ round friendship matrix using empirical data with the difference between initial friendship matrix and the simulation after 5 months. The proper *p* to best fit the difference was 0.2, which means that, on average every 5 weeks, one student changes one of her friendship connections in the network.

Fig 3(B) shows the comparison between correlation values of the empirical data and the simulation results. It indicates that the simulation graph after 5 months fits very well to the empirical data of the second round with *u = 0*.*023* which was relevant to a change in the brushing habit in about every 43 weeks on average.

### Model Results

**Fig 4:**
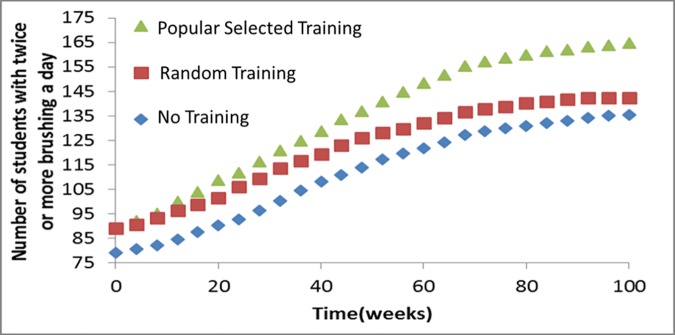
Simulation result of the three scenarios.

Simulation results of the three scenarios: “No Training”, “Random Training” and “Popular Selected Training” are shown in Fig 4. The diagram shows that, the number of students with the best level of dental brushing increased for all of the scenarios while the curves plateau almost after 100 weeks. Moreover, it indicates that training 10 popular students, resulted in an almost 30 more students to have the best brushing level compared to the “No training” scenario after 100 weeks, while educating 10 random students’ results of just about 5 students more than the “NO Training” scenario relatively.

## Discussion

Fig 2 shows the brushing frequency correlation value decreases as the friendship distance in the network increases. Moreover, the correlation length increases as students spend more time with each other, in the social network. This increment was observable after 5 months.

The most significant result of this study was brushing habits that actually spread over the network. At the beginning, the first year middle school students were not familiar with each other in past school years. The first assessment was performed in November, almost two months after the beginning of school year. Therefore, the friendship connections were fresh and formed very recently at the time. The second assessment was performed after 5 months in April. The increase in correlations of brushing habits among friends was indicative of behavior diffusion through the network. In addition, it showed that the system was not in a steady state during the period between the two assessments. This was a clear sign of network structure’s effect on the dynamics of brushing frequency among students. This means, during the time period between the two rounds (5 months), closer students in the network become more similar in their brushing habits. The ABM was designed to catch the growing correlation over time and **Fig 3** indicates that, the model with dynamic network was able to successfully explain the correlation length increment after 5 months. Moreover, these results indicate that, dental brushing behavior change, due to social pressure from friendship network, could be even observable in a couple of months.

As demonstrated in Table 3, when comparing the logistic regression models for the first and second rounds, the average level of brushing frequency among students has been improved within 5 months. This improvement can be explained by the fact that, there was a significant relation between the popularity of the agents with better brushing habits as demonstrated in Table 4. This may in turn encourage other students to improve their brushing behavior. Using dynamic network simulations, it also showed clear improvement in students’ dental brushing status even when there was no training (**Fig 4**: No Training scenario). This improvement can be described with behavior diffusion phenomenon.

Another possibility for this improvement’ reason is the effect of study design itself on the results. In this study, it is tried to minimize these effects. Given that students were unaware of being assessed again, Hawthorne effect [48] was unlikely to play a role in this study. In addition, students were assured that, the assessment results will not be used in any form, in favor or against them and their school-staff will not have any access to their questionnaire data. Furthermore, 11 to 12 years old students could hardly remember particularity of their answers to first round questions.

**Fig 4**, is a testing sample to show the power and limitations of the model when simulating different scenarios that may be considered by policy makers for assess the potential outcomes of their specific health promotion programs. In particular, it can demonstrate the outcome of any simple assumption considered. For example about the influence of training, the model was able to predict the results of employing different training scenarios.

Our simulation for the “No Training” scenario indicates that, the system gets into steady state, where the statistical characteristics don’t change any more, almost after 100 weeks. Actually, the increment of correlation range, from level 1 friendship to level 2, in the empirical rounds of assessments, indicated the dynamic characteristics of the system.

In this example, the model specifies that, “Popular Selected Training” scenario results in an increased number of students (compared to “No Training” scenario), with the best brushing level, of about three times more than the initially trained students. This result was remarkable when we observed that, randomly selected students for training resulted in an increased number of individuals (compared with “No Training” cases) with good of just half of the initially trained students.

Though, it seems obvious, the “Popular Selected” scenario had better results, but still there are many non-trivial quantitative points in the results which may be interesting for policy makers. For example:

1. It takes almost 100 weeks that the network becomes steady and the curves plateau in Fig 4.
2. After 100 weeks the “Popular Selected Training” scenario results in 30 students to have the best brushing level more than the “No Training” scenario. This value was 10 at the beginning, this means that 1 trained student results in 3 students with the highest brushing level after 100 weeks.
3. After 100 weeks the “Random Selected Training” scenario results in 5 students to have the best teeth brushing level which was more than the “No Training” scenario. This value was 10 at the beginning, this means that 1 trained student results in 0.5 student with the highest brushing level after 100 weeks.

Finally, it should be noted that, the proposed models, in contrast to diffusion models which consider Word of Mouth mechanism[49], did not rely on the promoting messages between peers in the network. The proposed model mostly relies on unintentional behavior learning among friends. This learning style can occur due to practical reasons like halitosis (bad breath) and/or nice smile (beautiful teeth).

## Conclusion

Oral health interventions are usually costly and require adequate resources. Due to lack of manpower, money or both many countries are facing oral diseases epidemic that has been referred as a major public health problem. In this investigation, it was shown that correlation length for brushing behavior improved after 5 months within the students’ social network connections in a school setting. Therefore, we can imply that oral health behavior improvement is possible through this noble methodology. This method requires no or minimum resources that can make almost all countries to use that.

When change can happen among students even within a short span of time, annual training plans may be the most cost-effective method of oral health behavior improvement for students’ populations through school settings. Identification and training of popular students can be considered as a short cut for achieving better outcomes in a shorter period of time.

The calibrated Agent Based Model is ready for policy makers to test any school based oral health promotion program scenarios in order to choose the best or most cost-effective ones.

## Supporting Information

S1 File Translated Questionnaire. (PDF)

S2 File Model source code. (RAR)

S1 Table Students’ SES and brushing frequency level data. (XLSX)
