# Supplementary material for: Friendship Network and Dental Brushing Behavior among Middle School Students: An Agent Based Modeling Approach
Source: PLoS One. 2017 Jan 19;12(1):e0169236. doi: 10.1371/journal.pone.0169236 (PMC5245796; doi:10.1371/journal.pone.0169236)
Supplement: S1 File — (PDF) [file pone.0169236.s001.pdf]

Dear Parents

One of the significant factors of public health is oral and dental public health. Poor oral health behaviors can have negative effects on students' functions in addition to their life style. Identification of the factors influencing oral health behavior can help policy makers to design proper promotion programs to improve oral and dental public health status. This questionnaire is designed to investigate some factors affecting oral health behaviors. We ask you to help us on this research. Your child has been invited to answer to the questionnaire. Please take whatever time you need to discuss the study with your family and friends, or anyone else you wish to. The decision to let your child join, or not to join, is up to you.

The questionnaire is designed to be answered by students but if they need, please help them for more accurate answers. The assessment results will not be used in any form, in favor or against students and their school-staff will not have any access to their questionnaire data.

Best regards

#	Questions
1	First Name and last name………….
2	Gender: Male… Female…..
3	Age in years: ………..
4	How often do you brush your teeth?<br>a) Twice in a day or more<br>b) Once a day<br>c) Some times in a week<br>d) Never
5	What is your mother's education level? (Can ask your mum)<br>a) Under high school diploma<br>b) High school Diploma<br>c) Academic degree

6	What is your father’s education level? (Can ask your mum)<br><br>a) Under high school diploma<br><br>b) High school Diploma<br><br>c) Academic degree
7	Do you have a family car<br><br>a) Yes<br><br>b) No
8	Do you have any of the following items at home?<br><br>a) Washing machine<br><br>b) Personal computer or laptop<br><br>c) Internet<br><br>d) Dishwasher<br><br>e) Microwave
9	Name at most five close friends among your classmates.
